# Tissue‐specific variation in nonsense mutant transcript level and drug‐induced read‐through efficiency in the *Cln1* ^*R151X*^ mouse model of INCL

**DOI:** 10.1111/jcmm.12744

**Published:** 2015-12-09

**Authors:** Vaughn Thada, Jake N. Miller, Attila D. Kovács, David A. Pearce

**Affiliations:** ^1^ Department of BiologyAugustana CollegeSioux FallsSDUSA; ^2^ Sanford Children's Health Research CenterSanford ResearchSioux FallsSDUSA; ^3^ Division of Basic Biomedical SciencesSanford School of Medicine of the University of South DakotaVermillionSDUSA; ^4^ Department of PediatricsSanford School of Medicine of the University of South DakotaSioux FallsSDUSA

**Keywords:** infantile neuronal ceroid lipofuscinosis, *Cln1*^*R151X*^, nonsense mutation, read‐through drug, ataluren

## Abstract

About 10% of inherited diseases are caused by nonsense mutations [*Trends Mol Med* **18** (2012) 688], and nonsense suppression drug therapy promoting translation through premature stop codons is an emerging therapeutic approach. Infantile neuronal ceroid lipofuscinosis (INCL), a childhood neurodegenerative disease, results from mutations in the *CLN1* gene encoding the lysosomal enzyme, palmitoyl‐protein thioesterase 1 (PPT1) [*Biochim Biophys Acta* **1832** (2013) 1806, *Hum Mutat* (2012) 63, *Biochim Biophys Acta* **1832** (2013) 1881]. The nonsense mutation p.R151X is the most common disease‐causing *CLN1* mutation *Hum Mutat* (2012) 63. In the novel *Cln1* ^*R151X*^ mouse model of INCL, we found large, tissue‐specific variations in *Cln1* ^*R151X*^ mRNA level and PPT1 residual enzyme activity. These tissue‐specific differences strongly influenced the read‐through efficiency of ataluren (PTC124), a well‐known nonsense suppression drug. A two‐day treatment with ataluren (10 mg/kg) increased PPT1 enzyme activity in the liver and muscle, but not in any other tissue examined. Our study identifies a new challenge/hurdle for read‐through drug therapy: variable efficiency of read‐through therapy in the different tissues/organs because of tissue‐specific variations in nonsense mutant transcript levels.

## Introduction

Nonsense mutations result in premature stop codons and are frequent causes of genetic diseases 1. mRNA transcripts containing premature stop codons are recognised and degraded by the nonsense‐mediated decay pathway to prevent the production and accumulation of truncated, non‐functional proteins within the cell 2. The so‐called read‐through drugs promote translation through premature stop codons, resulting full‐length proteins, which may be fully or partly functional, depending on the amino acid substitution for the stop codon. The first safe, specific and effective read‐through drug, PTC124, also known as ataluren, was described in a Nature Letter in 2007 3. The therapeutic effects of ataluren have been demonstrated in nonsense mutant mouse models of Duchene muscular dystrophy and cystic fibrosis 3, 4. Ataluren is an FDA‐approved investigational new drug, and it is currently in phase III clinical trials for cystic fibrosis (https://clinicaltrials.gov/ct2/show/NCT02139306) and for Duchenne muscular dystrophy (https://clinicaltrials.gov/ct2/show/NCT01826487).

Mutations in the *CLN1* gene cause a rare fatal childhood neurodegenerative disorder, infantile neuronal ceroid lipofuscinosis (INCL), also known as infantile Batten disease 5, 6. *CLN1* encodes a lysosomal enzyme, palmitoyl‐protein thioesterase 1 (PPT1), and the most frequent disease‐causing *CLN1* mutation is the p.R151X nonsense mutation 6, 7. Previously, we have shown that ataluren (PTC124) significantly elevates PPT1 enzyme activity in a patient‐derived INCL lymphoblast cell line, harbouring the p.R151X mutation in *CLN1* 8. To test if read‐through drugs represent a potential treatment for INCL, we have generated the *Cln1* ^*R151X*^ nonsense mutant mouse model of the disease. *Cln1* ^*R151X*^ mice show a dramatic decrease in *Cln1* mRNA level and CLN1 (PPT1) enzyme activity in the liver and brain, and display characteristic neuropathological and neurological features of the human disease 9. Treating *Cln1* ^*R151X*^ mice with ataluren multiple times per day dose‐dependently increased PPT1 protein level and enzyme activity in the liver and brain 9.

In genetic diseases caused by nonsense mutations usually multiple organs/tissues are affected, and it is assumed that for a particular disease read‐through drug therapy will provide comparable beneficial effects in various target organs/tissues. Tissue‐specific variations in the level of nonsense mutation‐containing transcripts, however, may strongly influence the efficacy of read‐through drugs. Supporting this notion, up to twofold differences in nonsense‐mediated mRNA decay efficiency in various murine tissues have been shown 10.

## Materials and methods

### Animals

*Cln1* ^*R151X*^ mice were maintained on a mixed 129S6/SvEv x C57BL/6J genetic background, and hybrid 129S6/SvEv x C57BL/6J mice served as WT controls. Mice were housed in individually vented microisolator cages (4–5 mice/cage) with *ad libitum* access to food and water. Mice were fed with the Teklad Global 2918 diet (Harlan Laboratories, Indianapolis, IN, USA), and their drinking water was tap water. All procedures were carried out according to the guidelines of the Animal Welfare Act and NIH policies, and were approved by the Sanford Research Animal Care and Use Committee.

### Sample processing and handling

For sample collection, mice were anesthetised followed by transcardial perfusion and vascular rinse using ice‐cold PBS. All tissue samples were collected and processed in a similar manner, and stored at −80°C no longer than 2 months before total RNA extraction or total protein isolation.

### Nucleic acid extraction

Total RNA was extracted from all tissue samples with a Maxwell 16 LEV simplyRNA Cells Kit (Promega, Madison, WI, USA) using a Maxwell 16 Instrument (Promega), according to the manufacturer's instructions. Sample purities and yields were determined using a Nanodrop Spectrophotometer (Thermo Fischer Scientific, Waltham, MA, USA). All samples had A_260_/A_280_ values between 2.03 and 2.20. RNA integrity was assessed as previously described 8.

### Reverse transcription

All samples, except muscle, were mass normalised using approximately 800 ng of total RNA for cDNA synthesis using a High Capacity cDNA Reverse Transcription Kit (Life Technologies, Carlsbad, CA, USA) according to the manufacturer's instructions in a 96‐well plate. Muscle samples were mass normalized using approximately 400 ng of total RNA. The reaction conditions were as follows: 25°C for 10 min., 37°C for 120 min., 85°C for 5 min. Samples were diluted with molecular grade water to 10 ng/μl following reverse transcription. Samples were assessed for DNA contamination using reactions with no reverse transcriptase added. All samples were DNA‐free and stored at −20°C until use.

### Quantitative real‐time polymerase chain reaction

Quantitative real‐time polymerase chain reaction (qPCR) was performed for the target gene using TaqMan hydrolysis assays (Life Technologies) for *Cln1* (Cat.# Mm00477078_m1). Quantitative real‐time polymerase chain reaction was performed for reference genes using TaqMan hydrolysis assays (Life Technologies) for *Gapdh* (Part.# Mm99999915_g1), *Hgprt* (Part.# 01318741_g1), *B2m* (Part.# Mm00437762_m1) and *Gusb* (Part.# Mm00446962_g1). Amplification was performed with 20 ng of cDNA in 10 μl reaction volumes for four technical replicates using Absolute Blue qPCR mix (Thermo Fischer Scientific) in 384 well plates (Roche Diagnostics, Indianapolis, IN, USA). Thermal cycling and fluorescence data collection were performed on a LightCycler 480 (Roche Diagnostics) using the following reaction conditions: 95°C for 15 min., followed by 40 cycles at 95°C for 15 sec., 60°C for 1 min.

### qPCR data analysis

Raw fluorescence data were analysed as previously described 8 using REST‐MCS software 11, 12.

### Protein isolation

Approximately 25–50 mg of each tissue sample was homogenised using an Ultra‐Turrax T8 homogenizer with a Dispergierstation T8.10 stand (Thermo Fischer Scientific) in 400 μl lysis buffer containing 50 mM Tris‐HCl, pH 7.4, 150 mM NaCl, 0.2% Triton X‐100 and 300 mM NP‐40, supplemented with 1:100 protease inhibitor cocktail and 1:1000 Phenylmethylsulfonyl fluoride (PMSF). This was followed by pulse sonication and 30 min. incubation on ice. Following a high‐speed centrifugation (12,000 × g at +4°C) for 10 min., the supernatant was collected and the total protein concentration was measured using the Pierce 660 nm Protein Assay (Thermo Fischer Scientific).

### PPT1 enzyme assay

Palmitoyl‐protein thioesterase 1 enzyme activity was measured in a fluorogenic enzyme assay as previously described 8, 9, 13, 14, 15, 16. Total protein samples isolated from *Cln1* ^*WT/WT*^ and *Cln1* ^*R151X/R151X*^ tissues were used. Ten micrograms of total protein per well (in a black 96‐well plate) was incubated in a mixture containing 0.64 mM of the fluorogenic PPT1 substrate, 4‐methylumbelliferyl‐6‐thiopalmitoyl‐β‐glucoside (MU‐6S‐palm‐βGlc; Moscerdam Substrates, Oegstgeest, the Netherlands), 15 mM dithiothreitol (DTT) in 5.1 mg/ml bovine serum albumin and 0.02% Na‐azide, 0.375% Triton X‐100 in 2/1 chloroform/methanol, 0.1 U β‐glucosidase from almonds (Sigma–Aldrich, St. Louis, MO, USA) in double‐concentrated McIlvains phosphate/citric‐acid buffer, pH 4.0. The total protein reaction mixture was then incubated for 1 hr at 37°C. The reaction was stopped with the addition of 0.5 M NaHCO_3_/0.5 M Na_2_CO_3_ buffer, pH 10.7 with 0.025% Triton X‐100 and fluorescence was measured using a SpectraMax M5 plate reader (Molecular Devices, Sunnyvale, CA, USA). Relative enzyme activity was estimated using total fluorescence minus background.

### Ataluren treatment

*Cln1* ^*R151X*^ male mice were randomly assigned to either a treatment group or vehicle control group. Eight mice were treated with 10 mg/kg ataluren (Selleck Chemicals, Houston, TX, USA) dissolved in PBS containing DMSO (2%) and (2‐hydroxypropyl)‐β‐cyclodextrin (22%) (Cat.# H5784; Sigma–Aldrich) *via* intraperitoneal (i.p.) injections four times daily for two consecutive days. Eight control mice were treated with the vehicle of the drug. Immediately following the last injection on the second day, tissues were collected and stored at −80°C for further use.

### Statistical analysis

The normality of all data sets was assessed using the Kolmogorov–Smirnov test and the Shapiro–Wilk test. All data were found to be normally distributed. Separate independent *t*‐tests were used to compare each tissue between control and ataluren‐treated mice. Levene's test for equality of variances was used to determine if variances of data sets being compared were equal. In instances where variances were found not to be similar (Levene's test statistic <0.05), unequal variances were assumed. All statistical analyses were done using IBM SPSS statistical software version 21 (IBM Corporation, Armonk, NY, USA) or GraphPad Prism software version 5.04 (GraphPad Software, Inc., La Jolla, CA, USA).

## Results

**Figure 1:**
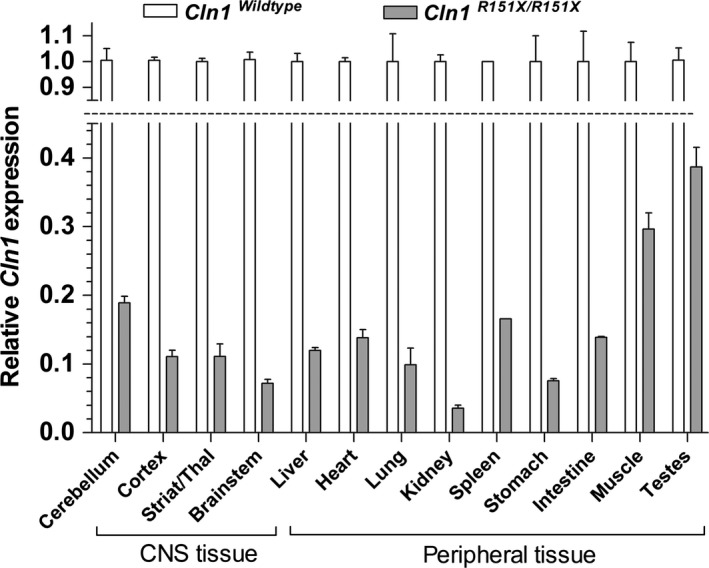
Tissue‐specific variations of *Cln1* mRNA level in *Cln1* ^*R151X*^ mice. Endogenous *Cln1* mRNA levels were measured by quantitative real‐time PCR in the brain (cerebellum, cortex, striatum/thalamus, brainstem) and peripheral tissues (liver, heart, lung, kidney, spleen, stomach, intestine, muscle, testes) from 5‐month‐old wild‐type (*white bars*) and *Cln1* ^*R151X*^ (*grey bars*) male mice. The relative expression of *Cln1* was normalized with four reference genes (*B2m*,* Gapdh*,* Gusb*,* Hgprt*). Columns and bars represent mean ± S.E.M. (three wild‐type and three *Cln1* ^*R151X*^ mice; four technical replicates were used for each mouse).

**Figure 2:**
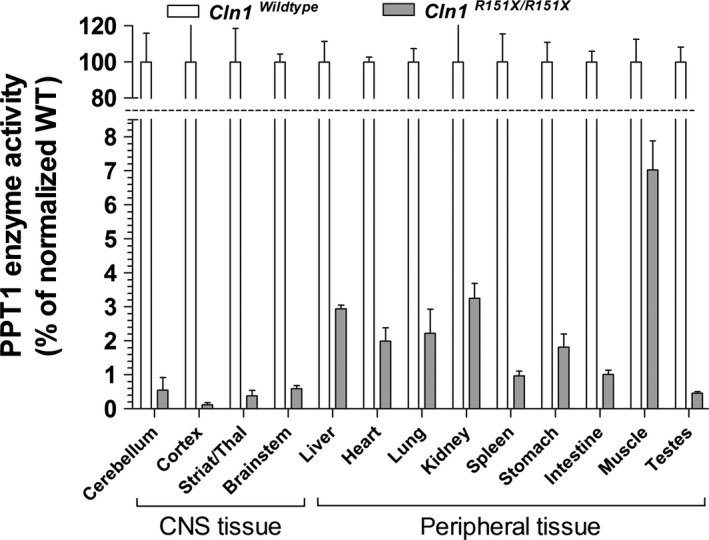
Tissue‐specific variations of PPT1 enzyme activity in *Cln1* ^*R151X*^ mice. Endogenous PPT1 enzyme activity was measured in the brain (cerebellum, cortex, striatum/thalamus, brainstem) and peripheral tissues (liver, heart, lung, kidney, spleen, stomach, intestine, muscle, testes) from 5‐month‐old wild‐type (*white bars*) and *Cln1* ^*R151X*^ (*grey bars*) male mice. The brain in particular has low levels of PPT1 activity compared to almost all peripheral tissues tested. Columns and bars represent mean ± S.E.M. (three wild‐type and three *Cln1* ^*R151X*^ mice; four technical replicates were used for each mouse).

To evaluate whether tissue‐specific differences in nonsense‐mediated decay affect the relative expression of *Cln1* mRNA and PPT1 enzyme activity in the *Cln1* ^*R151X*^ nonsense mutant mouse model of INCL, we compared multiple tissue types to each other. *Cln1* mRNA expression varied widely among tissues collected from the central nervous system and periphery, with the testes showing the highest level of *Cln1* expression (39% of wild‐type) and the kidney showing the lowest level of *Cln1* expression (~4% of wild‐type) (Fig. 1). Residual PPT1 enzyme activity, measured in a fluorogenic enzyme assay, also varied widely among tissues, with the muscle showing the highest level of PPT1 activity (7% of wild‐type) and the cerebral cortex showing the lowest level of PPT1 activity (0.1% of wild‐type) (Fig. 2). Interestingly, the brain, which is primarily affected in INCL, showed the lowest level of PPT1 activity overall. However, there was no significant correlation between *Cln1* mRNA expression and PPT1 enzyme activity in the tissues examined. This indicates that in addition to the strong effect of nonsense‐mediated mRNA decay on *Cln1* mRNA abundance, multiple layers of regulation, at both the mRNA and protein levels, affect the tissue‐specific expression of enzymatically active PPT1.

**Figure 3:**
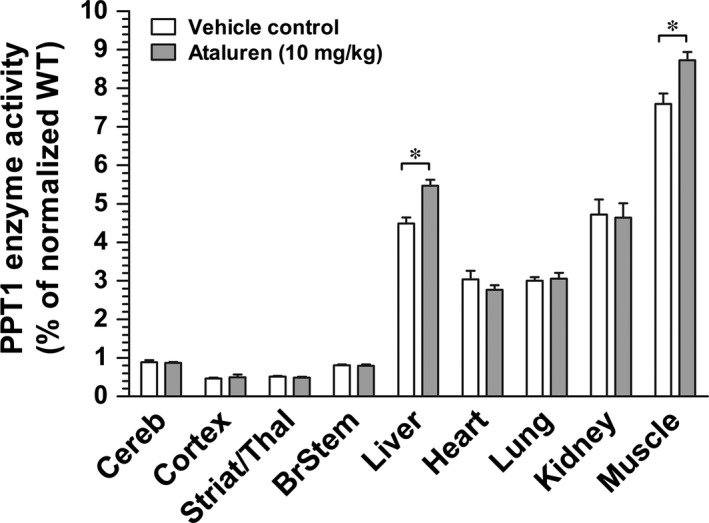
Tissue‐dependent effectiveness of the read‐through drug, ataluren (PTC124), in *Cln1* ^*R151X*^ mice. Two‐month old *Cln1* ^*R151X*^ male mice were treated with ataluren (10 mg/kg, i.p.) four times a day for 2 days. Control mice were treated with the vehicle of the drug. Immediately following the last injection on the second day, tissues were collected, and PPT1 enzyme activity was measured in the brain (cerebellum, cortex, striatum/thalamus, brainstem) and peripheral tissues (liver, heart, lung, kidney, skeletal muscle). Ataluren only increased PPT1 activity in the liver and muscle. Columns and bars represent mean ± S.E.M. (eight wild‐type and eight *Cln1* ^*R151X*^ mice). Statistical significance was determined by unpaired *t*‐test.: *p=0.004 for liver, *p=0.009 for skeletal muscle.

After finding tissue‐specific variations in *Cln1* mRNA level and PPT1 enzyme activity, we treated *Cln1* ^*R151X*^ mice with the read‐through drug, ataluren (10 mg/kg), injecting the drug intraperitoneally four times a day for two consecutive days. Immediately following the last injection on the second day, tissues were collected, and PPT1 enzyme activity was measured. This proof‐of‐concept trial was performed to identify tissue‐specific variations in the efficacy of read‐through therapy in the *Cln1* ^*R151X*^ mouse model of INCL. Ataluren treatment did not result in any significant increase in PPT1 enzyme activity in the different brain regions examined (cerebellum, cortex, striatum/thalamus and brainstem) (Fig. 3). Among the five peripheral tissues analysed from ataluren‐treated *Cln1* ^*R151X*^ mice, only the liver and skeletal muscle showed a slight but statistically significant increase in PPT1 enzyme activity as compared to the same tissues from control, vehicle‐treated mice (Fig. 3). The liver of ataluren‐treated and control (vehicle‐treated) *Cln1* ^*R151X*^ mice had 5.46% and 4.48% PPT1 activity, respectively (unpaired *t*‐test, *P* = 0.004). Skeletal muscle from ataluren‐treated *Cln1* ^*R151X*^ mice showed 8.72% PPT1 activity compared to 7.59% in the control group (unpaired *t*‐test, *P* = 0.009). Ataluren treatment did not increase PPT1 enzyme activity in the heart, lung and kidney (Fig. 3). Although the ~1% increase in PPT1 enzyme activity in the liver and muscle after a short‐term, 2‐day treatment, seems negligible, it has previously been demonstrated that restoring PPT1 activity to only 4–5% of the normal level results in significant therapeutic benefits 17.

## Discussion

Although nonsense‐mediated decay is an important process by which cells degrade mRNA transcripts containing premature stop codons to prevent the catastrophic damage of truncated proteins, very little is currently known about tissue‐specific nonsense‐mediated decay and how this could affect genetic disease pathology and therapeutic outcome 2. In the *Cln1* ^*R151X*^ nonsense mutant mouse model of INCL, we found large, tissue‐specific variations in *Cln1* ^*R151X*^ mRNA level, indicating tissue‐specific differences in nonsense‐mediated decay. Tissue‐specific variations in *Cln1* gene expression, however, might also contribute to the observed differences. Based on what is known about the nonsense‐mediated decay pathway and the degradation of truncated proteins within the cell, it is reasonable to conclude that if larger pools of *Cln1* ^*R151X*^ mRNA transcript are present, the more PPT1 enzyme activity will be measured because more transcripts are available for natural read‐through. This hypothesis is not supported, however, when the tissue‐specific *Cln1* mRNA levels and PPT1 enzyme activities are compared. Although skeletal muscle has both the highest levels of *Cln1* mRNA expression and PPT1 enzyme activity, kidney has the lowest *Cln1* mRNA level, yet the second highest level of PPT1 enzyme activity. Complete analysis of *Cln1* mRNA level as it relates to PPT1 enzyme activity in the same tissue yielded no correlation. The lack of correlation could be because of tissue‐specific differences in the level of *Cln1* mRNA synthesis and rate of PPT1 protein turnover. Since premature stop codons frequently induce alternative splicing 18, 19, another possible explanation is that the *Cln1* ^*R151X*^ mutation‐induced altered splicing is different in the various tissues. The known tissue‐specific differences in the unfolded protein response 20 in combination with the recently identified negative regulation of unfolded protein response by NMD 21 could also contribute to the observed lack of correlation between *Cln1* mRNA level and PPT1 enzyme activity in tissues of *Cln1* ^*R151X*^ mice.

Treatment of *Cln1* ^*R151X*^ mice with the read‐through drug, ataluren (10 mg/kg), resulted in a slight but statistically significant increase in PPT1 enzyme activity in the liver and muscle only. Although the ~1% increase in PPT1 enzyme activity in the liver and muscle after a short‐term, 2‐day treatment, seems negligible, it has previously been demonstrated that restoring PPT1 activity to only 4–5% of the normal level results in significant therapeutic benefits 17. The current experiment involved administering ataluren for only two days. The lack of increased PPT1 enzyme activity in the heart, lung, brain and kidney may suggest that more time is required for ataluren to achieve optimal concentrations in some tissues. In a previous study, we have shown that much higher dose of ataluren (100 mg/kg) is required to increase PPT1 enzyme activity within the brain 9. With this higher dose of ataluren (100 mg/kg), however, the therapeutic effect in the liver was lost 9, indicating that above a certain tissue concentration ataluren does not promote translation through the premature stop codon.

In summary, our study identified a new challenge/hurdle for read‐through drug therapy: variable efficiency of read‐through therapy in the different tissues/organs because of tissue‐specific variations in nonsense mutant transcript levels. This means that a read‐through drug at any given dose cannot be therapeutically effective in all tissues. Accordingly, it will be important to consider which organ is affected the most in a particular genetic disease and to determine the optimal dose of read‐through drug therapy for that organ.

## Conflicts of interest

The authors declare no conflicts of interest.
